# Evaluating multimodal AI in medical diagnostics

**DOI:** 10.1038/s41746-024-01208-3

**Published:** 2024-08-07

**Authors:** Robert Kaczmarczyk, Theresa Isabelle Wilhelm, Ron Martin, Jonas Roos

**Affiliations:** 1 Department of Dermatology and Allergy, School of Medicine, Technical University of Munich, Munich, Germany; 2 Eye Center, Faculty of Medicine, Albert-Ludwigs-University of Freiburg, Freiburg, Germany; 3 Clinic of Plastic, Hand and Aesthetic Surgery, Burn Center, BG Clinic Bergmannstrost, Halle (Saale), Germany; 4 Department of Orthopedics and Trauma Surgery, University Hospital of Bonn, Bonn, Germany

**Keywords:** Medical research, Signs and symptoms

## Abstract

This study evaluates multimodal AI models’ accuracy and responsiveness in answering NEJM Image Challenge questions, juxtaposed with human collective intelligence, underscoring AI’s potential and current limitations in clinical diagnostics. Anthropic’s Claude 3 family demonstrated the highest accuracy among the evaluated AI models, surpassing the average human accuracy, while collective human decision-making outperformed all AI models. GPT-4 Vision Preview exhibited selectivity, responding more to easier questions with smaller images and longer questions.

## Multimodal AI for medical diagnosis: potential and challenges

The rapid integration of Large Language Models (LLMs) like GPT-4 into various domains necessitates their evaluation in specialized tasks such as medical diagnostics^1–3^.

Recent studies evaluating the viability of GPT-4V and similar models have demonstrated their potential to augment human expertise in clinical settings^1^. These advances promise transformative potential, e.g. streamlining access to medical diagnostics. However, they also pose challenges regarding reliability and raise ethical concerns^4^. Moreover, the rise of multimodal capabilities of LLMs requires reevaluating their abilities beyond just textual contexts and the interpretation of clinical questions. A recent study evaluating the multimodal performance in radiology has shown that the detection of pathology in radiologic imaging is still inaccurate^5^. However, this analysis was only performed for GPT-4V, so no overall statement can be made about the performance of multimodal LLMs. The diagnostic process in the presented NEJM case studies, on the other hand, is more complex and diverse than single-specialty radiologic interpretation and requires the integration of different information. Previous studies on this topic have shown mixed results regarding the diagnostic accuracy of large language models. The prompting, the model used, the specialty and the specific dataset used appear to have an influence on the results. This leads to an instability of the results and is currently a limitation of the evaluation of the diagnostic accuracy of these models^6,7^. With the introduction of image analysis capabilities, there is now the opportunity to provide these models with additional information and create a more realistic representation of medical cases^8^.

## NEJM image challenge dataset

Our data shows high participation in the NEJM Image Challenge that started on the 13th of October 2005, culminating in over 85 million responses to 945 cases (as of 13th December 2023). The mean response count per question was 90,679 (SD = 32,921; median = 88,407; range = 13,120–233,419). The average percentage of votes that answered the medical cases correctly was 49.4% (SD = 13.6%; median = 49%; range = 16–88%), reflecting the diverse difficulty levels inherent in the case questions. The length of questions ranged from 4 to 128 words with 28.5 words on average, indicating a varied scope of additional clinical information provided. The medical images analyzed in the NEJM Image Challenge exhibited a broad range in resolution, with sizes varying from 0.57 to 5.95 megapixels. On average, the images were 2.02 megapixels, suggesting a substantial diversity in image detail and quality presented to the AI models and the public for interpretation.

## Responsiveness

**Fig. 1:**
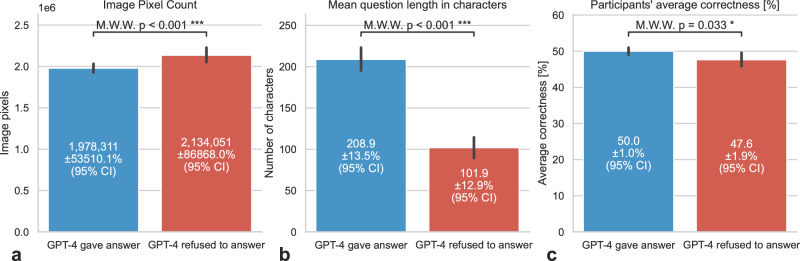
GPT-4V Answer Status vs image pixel count, question length, and participants’ average correctness. This bar plot illustrates the image mean pixel count (**a**), the mean question length measured in characters (**b**) and the participants’ average correctness (**c**) for questions with 95% confidence intervals where GPT-4V provided an answer compared to those where GPT-4V refused to answer. The data indicate that GPT-4V was more likely to give answers to easier questions, as evidenced by a higher average correctness among participants. Specifically, participants had an average correctness of 50.0% (±1.0% 95% CI) for questions answered by GPT-4V, compared to 47.6% (±1.9% 95% CI) for questions where GPT-4V did not provide an answer (*p* = 0.033). Moreover, the images in answered questions tend to have less pixels (*p* < 0.001) and the questions measured in number of characters are longer (*p* < 0.001). We have utilized the two-sided Mann-Whitney *U*-Test.

While all open-source models in addition to the proprietary models of Anthropic’s Claude 3 family responded to all queries, the proprietary GPT-4 Vision Preview (e.g., “I’m sorry, I cannot provide medical diagnoses or interpret medical images. […]”) responded to only 76% (*n* = 718) of cases. GPT-4 Vision Preview was more inclined to answer easier questions measured by the human participants average correctness (*p* = 0.033), as well as questions with smaller image sizes (*p* < 0.001) and longer question texts (*p* < 0.001, Fig. 1). Bard Gemini 1.0 Vision Pro only failed to give a response to one question (0.11%) out of unknown reasons (“block_reason: OTHER”).

## Accuracy

**Fig. 2:**
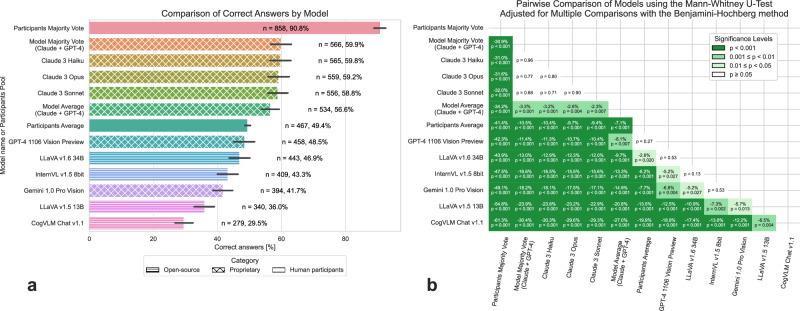
Accuracy of multimodal models in medical image analysis. Comparison of a wide variety of multimodal models both, open-source models and proprietary models against the participants average and majority vote in the multiple-choice NEJM Image Challenge of 945 cases. The error bars depict 95% confidence intervals of the mean (**a**). The heatmap shows the pairwise comparisons of models and participants using the two-sided Mann-Whitney *U*-Test, with *p*-values adjusted for multiple comparisons using the Benjamini-Hochberg method. The mean difference in correct answers is annotated for significant comparisons, with *p*-values displayed underneath (**b**). Significance levels are indicated by color: dark green (*p* < 0.001), middle green (0.001 ≤ *p* < 0.01), light green (0.01 ≤ *p* < 0.05), and white (*p* ≥ 0.05). Except for GPT-4 1106 Vision Preview, that has only answered 76% of the questions (*n* = 718) and Gemini 1.0 Vision Pro answering all questions but one, all questions were answered by the participants and the other models. The questions that were not answered by GPT-4V and Gemini 1.0 Vision Pro were considered not correct.

Among the AI models, Anthropic’s models stood out, achieving the highest accuracy (between 58.8%, *n* = 556 out of 945 to 59.8%, *n* = 565 out of 945 questions) greatly surpassing the participants average vote (49.4%, *p* < 0.001) by around 10%. We observed that the collective human decision, determined by majority vote with 7 ties counted as incorrect responses, answered 90.8% (*n* = 858) of cases correctly, revealing the capabilities of swarm intelligence in medical multimodal diagnostics und surpassing all tested multimodal models by a great margin (Fig. 2). The model majority vote that considered the four best models (all Claude 3 models in addition to GPT-4 1106 Vision Preview) has not shown any improvements (*p* = 0.96) to the best model Claude 3 Haiku. Interestingly, Haiku is the smallest and fastest model of the Claude 3 family and answered six questions more correctly compared to the largest, most capable model Opus, though the difference was not statistically significant (*p* = 0.8).

## GPT-4V selectivity in answering questions

This discernment in response behavior by OpenAI’s flagship model^9^ underscores the potential limitations imposed by restrictive moderation policies on closed-source AI models^10,11^. Such selectivity, particularly when most clinical questions receive a response despite moderation, calls into question the effectiveness of these restrictive measures and their influence on scientific evaluation. Interestingly, this behavior seems to be selective to towards easier questions, questions with smaller images and questions containing longer text descriptions. To enhance transparency, developers should clearly articulate the reasons behind the non-responsiveness to certain inquiries (e.g., inappropriate image material or privacy concerns for non-anonymized patient photos, or simply the alignment of the model to not be wrong and rather not answering a question than answering it falsely). Introducing specialized accounts for researchers with expanded access rights and less restrictive models could be a beneficial approach to support research in this field, while ensuring compliance with ethical and security standards.

These observations show promising AI capabilities and limitations, such as erroneousness and restrictive responsiveness, in the medical multimodal domain when moving beyond purely textual analysis, a setting where AI has exceptionally performed in surpassing human capabilities in several studies^1^.

## AI capabilities in medical diagnostics

All Claude 3 models surpass OpenAI’s GPT-4 Vision Preview in terms of correctness without denying any questions, which might indicate for better aligned training methods at Anthropic. In general, we have showed that general purpose models are very well suited to answer highly specific medical knowledge questions, and even surpassing the participant’s average correctness. In our study, Claude 3 Haiku achieved the highest accuracy. Similar results were observed in another study where text-only GPT-4 outperformed 99.98% of simulated human readers—although comprising only 38 cases—in diagnosing NEJM cases^1^, a result not replicated in our multi-modal image challenge analysis using GPT-4 Vision Preview. Human collective intelligence surpassed all AI models with a 90.8% accuracy rate, aligning with the concepts outlined by James Surowiecki^12^. Overall, the findings are promising for the future of AI in medical diagnostics, particularly in areas like dermatology where the automation of cancer detection is showing increasing scientific interest. A recently published meta-analysis demonstrated that the accuracy of AI in detecting skin cancer significantly exceeded that of general practitioners and showed comparable performance to experienced dermatologists^13^. Furthermore, another analysis found that an accuracy of over 90% in skin cancer detection could be achieved using AI models^14^. These results suggest that the analytical capabilities of AI for specific diagnostic tasks, such as skin cancer detection, significantly exceed those observed in our more general multimodal analysis. It has been shown that safety mechanisms designed to prevent medical self-diagnosis by non-professionals are inadequate due to inconsistent implementation^15^. The findings from our study and others indicate that while AI can significantly support medical diagnosis and training and streamline medical access, its integration into clinical practice requires a cautious, conscientious, and transparent approach^1,4,5,16^ with imperative regulatory oversight^2,3^.

## Transparency and EU regulatory landscape

Just recently, the EU Parliament passed the EU AI Act, a landmark legislation that aims to regulate artificial intelligence by categorizing AI applications based on their risk levels^17^. The Act places stringent requirements on high-risk AI systems, including those used in healthcare. This regulation mandates transparency, robustness, and human oversight, ensuring that AI systems operate safely and ethically. For medical AI, the EU AI Act emphasizes the necessity for clear documentation, traceability, and accountability of AI decision-making processes. It also underscores the importance of rigorous testing and validation to meet high standards of accuracy and reliability. Open models analyzed in our study have a clear advantage here because they have openly available model weights and usually good documentation of training code and datasets used, facilitating transparency and traceability as required by the EU AI Act.

## Evaluation challenges and future research direction

The evaluated multimodal models are not custom designed for medical tasks, and while their performance is promising and strong transfer learning has been shown for general purpose models^18^, the study of specialized, fine-tuned large language models is warranted. Clinical trials are essential to validate the capabilities of multimodal AI in clinical routines. Additionally, the proprietary models lack a comprehensive safety review due to inaccessible training datasets and model architectures. The lack of transparency concerning the training data of proprietary models casts uncertainty on whether this evaluation qualifies as a true zero-shot scenario, suggesting possible “dataset contamination” where the images or questions might have been included in the models’ training datasets. Conversely, the transparency of open-source models may facilitate more robust safety evaluations. Furthermore, the structured nature of multiple-choice formats may not fully capture the complexities encountered in real-world clinical settings, where diagnoses are not confined to predetermined options. Finally, it’s noteworthy that a single model, when configured with varying parameters, can yield different responses. In our study, each model was utilized without any parameter adjustments to evaluate the base capabilities.

As AI models rapidly evolve, they offer substantial promise in augmenting medical diagnostics, extending their potential beyond the traditionally text-centric applications to include multimodal datasets. Yet, our findings endorse a tempered optimism and call for a nuanced appreciation of these tools’ capabilities. Establishing robust frameworks for responsible deployment is crucial for patient safety^3^. The future of AI in medicine depends on collaborative efforts to enhance its reliability and ethical application, with the goal of complementing—rather than replacing—human expertise.

## Methods

### Data and variables

The data was derived from New England Journal of Medicine’s (NEJM) image challenge^19^, a weekly web quiz that contains an image, an optional short case description, a corresponding question and five multiple-choice questions. All image cases published until the 7th of December 2023 were included (*n* = 945). In addition to the above question, the number of votes for the available options was also obtained to compare the models against human collective intelligence.

Two metrics were derived from participants’ voting data: the participants’ mean, representing the average percentage of people who answered each question correctly, and the participant’s majority vote, determining whether most participants selected the correct answer for each question, serving as a metric of collective consensus on the correctness of responses.

### Multimodal models and question prompt

The present study evaluates nine multimodal AI models: CogVLM Chat v1.1^20^, LLaVA v1.5 13B^10^, LLaVA v1.6 34B^21^, InternVL-Chat-V1.5-Int8^22^, OpenAI’s GPT-4 Vision Preview v1106^23^ and Google’s Gemini 1.0 Pro Vision^24^ and Anthropic’s Claude 3 Family Opus, Haiku and Sonnet^25^. The proprietary GPT-4 Vision Preview, Gemini 1.0 Pro Vision and Claude 3 models were used through the company’s python libraries^23,24,26^. The model weights of the open models were downloaded from Hugging Face^27^ on the 18th of December, 2024 except for LLaVA v1.6 34B and InternVL-Chat-V1.5-Int8, which were accessed on the 12th and the 19th of May, 2024 respectively.

The same question template was used for all nine multimodal models (Box 1).

Box 1 Prompt template used for all models Act as an expert physician and professor at a renowned university hospital. Your task is to answer medical questions, primarily based on descriptions of medical images. Use your expertise to interpret these descriptions accurately and provide the most likely diagnosis or answer. <OPTIONAL-CASE-DESCRIPTION > <CASE-QUESTION>

A) < OPTION-A>

B) < OPTION-B>

C) < OPTION-C>

D) < OPTION-D>

E) < OPTION-E>

Provide the answer to the multiple choice question in the format: <correct_letter > ) <correct_answer > . Include a brief explanation if possible to support the answer.

### Statistical analysis

The analysis was conducted on an Apple M1 Pro macOS 14.3.1 system, using Python 3.10.12. We used several Python libraries for data analysis and visualization: Pandas (v1.5.3) for data manipulation, Seaborn (v0.11.2) and Matplotlib (v3.7.2) for generating plots.

### Declaration of generative AI and AI-assisted technologies in the writing process

Grammarly (Grammarly, Inc.) and GPT-4 were used for language improvements and general manuscript revision. After using these tools, the authors reviewed and edited the content as needed and take full responsibility for the publication’s content.

### Reporting summary

Further information on research design is available in the Nature Portfolio Reporting Summary linked to this article.

### Supplementary information

Supplementary Information

Reporting summary checklist

Supplementary Data 1

## Data Availability

All model responses in this study are documented and uploaded as a tab delimited file, ensuring transparency and reproducibility of our findings (Supplementary Data 1). The NEJM Image Challenge cases are openly accessible without the need for login at the New England Journal of Medicine’s Image Challenge website NEJM Image Challenge^19^. This public availability of the full raw dataset supports further research and scrutiny by the medical and scientific communities.

## Code availability

The source code for the interactive Python Jupyter notebook used in the analysis of this study is available on reasonable request. Requests for access or clarification can be directed to the corresponding author.
