# Supplementary material for: Evaluating multimodal AI in medical diagnostics
Source: NPJ Digit Med. 2024 Aug 7;7:205. doi: 10.1038/s41746-024-01208-3 (PMC11306783; doi:10.1038/s41746-024-01208-3)
Supplement: Supplementary file 1 — Supplementary Information [file 41746_2024_1208_MOESM1_ESM.pdf]

Supplementary Data 1: Responses for all multimodal models

The file "model_responses.tsv" is a tab-delimited file containing the following columns: **id**: Unique identifier for each entry representing the NEJM image challenge, that can be accessed through "*https://www.nejm.org/image-challenge?ci=id*", **model_name**: name of the multimodal model used, **model_response**: the response generated by the model, **correct_answer**: The correct answer for the given query (A – E), **response_letter**: The letter associated with the model's response (A – E), the complete response was provided wherever the model was not responding with an answer, **answer_type**: The type or category of the answer (either ANSWER or NOANSWER), **correct**: indicates whether the model's response is correct or not (boolean: true or false). The models involved are 'internvl-1.5-8bit', 'gemini-pro-vision', 'cogvlm-chat-hf-chat-template', 'claude-3-sonnet-20240229', 'llava-1.5-13b-hf', 'claude-3-haiku-20240307', 'llava-34b-v1.6-fp16', 'claude-3-opus-20240229', and 'gpt-4-1106-vision-preview'.
