# Supplementary material for: Evaluating multimodal AI in medical diagnostics
Source: NPJ Digit Med. 2024 Aug 7;7:205. doi: 10.1038/s41746-024-01208-3 (PMC11306783; doi:10.1038/s41746-024-01208-3)
Supplement: Supplementary file 2 — Reporting summary checklist [file 41746_2024_1208_MOESM2_ESM.pdf]

nature portfolio

Corresponding author(s): Theresa Isabelle Wilhelm

Last updated by author(s): Jun 4, 2024

Reporting Summary

Nature Portfolio wishes to improve the reproducibility of the work that we publish. This form provides structure for consistency and transparency in reporting. For further information on Nature Portfolio policies, see our Editorial Policies and the Editorial Policy Checklist.

Statistics

For all statistical analyses, confirm that the following items are present in the figure legend, table legend, main text, or Methods section.

n/a	Confirmed	
☐	☒	The exact sample size (n) for each experimental group/condition, given as a discrete number and unit of measurement
☒	☐	A statement on whether measurements were taken from distinct samples or whether the same sample was measured repeatedly
☐	☒	The statistical test(s) used AND whether they are one- or two-sided<br>*Only common tests should be described solely by name; describe more complex techniques in the Methods section.*
☒	☐	A description of all covariates tested
☐	☒	A description of any assumptions or corrections, such as tests of normality and adjustment for multiple comparisons
☐	☒	A full description of the statistical parameters including central tendency (e.g. means) or other basic estimates (e.g. regression coefficient) AND variation (e.g. standard deviation) or associated estimates of uncertainty (e.g. confidence intervals)
☐	☒	For null hypothesis testing, the test statistic (e.g. F, t, r) with confidence intervals, effect sizes, degrees of freedom and P value noted<br>*Give P values as exact values whenever suitable.*
☒	☐	For Bayesian analysis, information on the choice of priors and Markov chain Monte Carlo settings
☒	☐	For hierarchical and complex designs, identification of the appropriate level for tests and full reporting of outcomes
☒	☐	Estimates of effect sizes (e.g. Cohen's d, Pearson's r), indicating how they were calculated

Our web collection on statistics for biologists contains articles on many of the points above.

Software and code

Policy information about availability of computer code

Data collection	The data was derived from New England Journal of Medicine's (NEJM) image challenge8, a weekly web quiz that contains an image, an optional short case description, a corresponding question and five multiple-choice questions. All image cases published until the 7th of December 2023 were included (n=945). In addition to the above question, the number of votes for the available options was also obtained to compare the models against human collective intelligence.
Data analysis	Two metrics were derived from participants' voting data: the participants' mean, representing the average percentage of people who answered each question correctly, and the participant's majority vote, determining whether most participants selected the correct answer for each question, serving as a metric of collective consensus on the correctness of responses. The analysis was conducted on an Apple M1 Pro macOS 14.3.1 system, using Python 3.10.12. We used several Python libraries for data analysis and visualization: Pandas (v1.5.3) for data manipulation, Seaborn (v0.11.2) and Matplotlib (v3.7.2) for generating plots.

For manuscripts utilizing custom algorithms or software that are central to the research but not yet described in published literature, software must be made available to editors and reviewers. We strongly encourage code deposition in a community repository (e.g. GitHub). See the Nature Portfolio guidelines for submitting code & software for further information.

Data

Policy information about availability of data

All manuscripts must include a data availability statement. This statement should provide the following information, where applicable:

- Accession codes, unique identifiers, or web links for publicly available datasets
- A description of any restrictions on data availability
- For clinical datasets or third party data, please ensure that the statement adheres to our policy

All model responses in this study are documented and uploaded as a tab delimited file, ensuring transparency and reproducibility of our findings. The NEJM Image Challenge cases are openly accessible without the need for login at the New England Journal of Medicine's Image Challenge website NEJM Image Challenge8. This public availability supports further research and scrutiny by the medical and scientific communities.

Research involving human participants, their data, or biological material

Policy information about studies with human participants or human data. See also policy information about sex, gender (identity/presentation), and sexual orientation and race, ethnicity and racism.

Reporting on sex and gender	Sex- and gender-based analyses were not performed, as the participants on the NEJM Image Challenge are anonymous (without knowing the sex or gender).
Reporting on race, ethnicity, or other socially relevant groupings	No analysis for race, ethnicity or other socially relevant groupings (see above).
Population characteristics	See above.
Recruitment	See above.
Ethics oversight	See above (use of secondary data).

Note that full information on the approval of the study protocol must also be provided in the manuscript.

Field-specific reporting

Please select the one below that is the best fit for your research. If you are not sure, read the appropriate sections before making your selection.

☒ Life sciences ☐ Behavioural & social sciences ☐ Ecological, evolutionary & environmental sciences

For a reference copy of the document with all sections, see nature.com/documents/nr-reporting-summary-flat.pdf

Life sciences study design

All studies must disclose on these points even when the disclosure is negative.

Sample size	All multiple-choice questions to the cutoff date were taken from the NEJM Image Challenge (n = 945).
Data exclusions	No data was excluded.
Replication	The data is freely available, and we provide the code and prompts for reproducibility.
Randomization	No randomization was necessary, as the data is secondary in nature and only experiments with Large Language Models were utilized.
Blinding	Not necessary, see also 'Randomization'.

Reporting for specific materials, systems and methods

We require information from authors about some types of materials, experimental systems and methods used in many studies. Here, indicate whether each material, system or method listed is relevant to your study. If you are not sure if a list item applies to your research, read the appropriate section before selecting a response.

Materials & experimental systems

n/a	Involved in the study
☒	☐ Antibodies
☒	☐ Eukaryotic cell lines
☒	☐ Palaeontology and archaeology
☒	☐ Animals and other organisms
☒	☐ Clinical data
☒	☐ Dual use research of concern
☒	☐ Plants

Methods

n/a	Involved in the study
☒	☐ ChIP-seq
☒	☐ Flow cytometry
☒	☐ MRI-based neuroimaging

Plants

Seed stocks

Report on the source of all seed stocks or other plant material used. If applicable, state the seed stock centre and catalogue number. If plant specimens were collected from the field, describe the collection location, date and sampling procedures.

Novel plant genotypes

Describe the methods by which all novel plant genotypes were produced. This includes those generated by transgenic approaches, gene editing, chemical/radiation-based mutagenesis and hybridization. For transgenic lines, describe the transformation method, the number of independent lines analyzed and the generation upon which experiments were performed. For gene-edited lines, describe the editor used, the endogenous sequence targeted for editing, the targeting guide RNA sequence (if applicable) and how the editor was applied.

Authentication

Describe any authentication procedures for each seed stock used or novel genotype generated. Describe any experiments used to assess the effect of a mutation and, where applicable, how potential secondary effects (e.g. second site T-DNA insertions, mosiacism, off-target gene editing) were examined.
